# Gold Nanorods for Doxorubicin Delivery: Numerical Analysis of Electric Field Enhancement, Optical Properties and Drug Loading/Releasing Efficiency

**DOI:** 10.3390/ma15051764

**Published:** 2022-02-26

**Authors:** Muhammad Qamar, Ghulam Abbas, Muhammad Afzaal, Muhammad Y. Naz, Abdul Ghuffar, Muhammad Irfan, Stanislaw Legutko, Jerzy Jozwik, Magdalena Zawada-Michalowska, Abdulnour Ali Jazem Ghanim, Saifur Rahman, Usama M. Niazi, Mohammed Jalalah, Fahad Salem Alkahtani, Mohammad K. A. Khan, Ewelina Kosicka

**Affiliations:** 1 Department of Physics, Riphah International University Faisalabad Campus, Faisalabad 44000, Pakistan; qamarjutt707@gmail.com (M.Q.); muhammad.afzaal@riphahfsd.edu.pk (M.A.); ghuffar2628@gmail.com (A.G.); 2 Department of Physics, University of Agriculture Faisalabad, Faisalabad 38040, Pakistan; yasin306@uaf.edu.pk; 3 Electrical Engineering Department, College of Engineering, Najran University Saudi Arabia, Najran 11001, Saudi Arabia; miditta@nu.edu.sa (M.I.); srrahman@nu.edu.sa (S.R.); msjalalah@nu.edu.sa (M.J.); fsalkhtani@nu.edu.sa (F.S.A.); 4 Faculty of Mechanical Engineering, Poznan University of Technology, 60-965 Poznan, Poland; stanislaw.legutko@put.poznan.pl; 5 Faculty of Mechanical Engineering, Lublin University of Technology, 20-618 Lublin, Poland; j.jozwik@pollub.pl (J.J.); m.michalowska@pollub.pl (M.Z.-M.); e.kosicka@pollub.pl (E.K.); 6 Civil Engineering Department, College of Engineering, Najran University Saudi Arabia, Najran 61441, Saudi Arabia; aaghanim@nu.edu.sa; 7 Department of Mechanical Engineering Technology, National Skills University Islamabad, Islamabad 44000, Pakistan; ukniaxi@gmail.com; 8 Mechanical Engineering Department, College of Engineering, Najran University Saudi Arabia, Najran 11001, Saudi Arabia; mkkhan@nu.edu.sa

**Keywords:** COMSOL Multiphysics, gold nanorods, doxorubicin, drug delivery

## Abstract

The optical properties and electric field enhancement of gold nanorods for different cases were investigated in this study. The numerical analysis was carried out to understand the functionality and working of gold nanorods, while the experimental portion of the work was focused on the efficiency of gold nanorods for targeted drug delivery. COMSOL Multiphysics was used for numerical analysis. The theoretical results suggest the use of gold nanorods (AuNRs) for anticancer applications. The resonance peaks for gold nanorods of 10 nm diameter were observed at 560 nm. The resonance peaks shifted towards longer wavelengths with an increase in nanorod size. The resonance peaks showed a shift of 140 nm with a change in nanorod length from 25 to 45 nm. On the experimental side, 22 nm, 35 nm and 47 nm long gold nanorods were produced using the seed-mediated growth method. The surface morphology of the nanorods, as well as their optical characteristics, were characterized. Later, gold nanorods were applied to the targeted delivery of the doxorubicin drug. Gold nanorods showed better efficiency for doxorubicin drug loading time, release time, loading temperature, and release temperature. These results reveal that AuNRs@DA possess good ability to load and deliver the drug directly to the tumorous cells since these cells show high temperature and acidity.

## 1. Introduction

The optical characteristics of gold nanoparticles (AuNPs) are different from their bulk materials. The morphology of individual particles in nanomaterials determines their chemical, biological and physical properties [1,2]. When the size of a gold nanorod (AuNR) is smaller than the operational wavelength of incident light, it strongly interacts with photon energy. The result is the production of extra oscillating electrons in atoms and sub-waves. In the nanorods, the oscillating mode creates localized surface plasmon resonance (LSPR) [3]. The main applications of nanorods are vast, ranging from optics, medicine, biomedical devices, renewable energy devices, microelectronic, and biomedical imaging [4].

Gold (Au) nanocrystal shape is also a critical factor in determining its chemical and physical properties. The topological features of Au nanocrystals affect the anisotropic electrical and optical responses more than those of spherical nanocrystals [5]. Gold nanorods and gold nanospheres are used as contrast agents for tumor imaging. Gold nanorods are assumed better imaging agents than gold nanospheres. By using the nanorods, the absorption of light by the organic molecules can be limited to the near infrared region, which make it easy to distinguish the gold signals [6]. The Raman signal of assembled gold nanorods is much greater than that of nanospheres [2].

LSPR is the most exciting feature of Au nanocrystals. It is based on the collective oscillation of free electrons at the nanoscale [7]. Au nanocrystals possess a special light focusing capacity. They show good ability to focus the free-space optical field in subwavelength areas under resonant simulation close to their surfaces [8,9]. These nanocrystals have a unique ability to generate extremely strong electric fields around them, allowing for an array of light-matter interactions having efficient procedures, such as high-harmonic generation [10], plasmon-enhanced spectroscopies [11], plasmon-induced vacuum Rabi splitting [12], optical nanoantenna effects [13], plasmon-assisted photochemical reactions [14] and photothermal conversion [15].

Several well-known methods are being used to manufacture gold nanoparticles [16]. In the context of synthesis, gold salts are frequently reduced to nanoparticles when a reducing agent is added in the reaction. In most situations, stabilizing agents are employed to balance the dispersion. They bond to the nanoparticles’ surfaces and regulate particle size. It is usual to apply citric acid for the reduction of gold salts to NPs [17]. Collective oscillations occur when free electrons in nanoparticles receive energy from light [18]. The absorbed light induces heat inside the nanoparticles because the absorption cross-section of AuNPs is large at the plasmon resonance frequency [19]. The absorbed light warms the particles and distributes the heat to the rest of the surroundings [20,21,22,23]. The development of methods for obtaining liquid dispersions of rod-shaped gold nanoparticles has progressed. The template synthesis [24], electrochemical synthesis [25], and seed-mediated growth [26] techniques are available to produce gold nanorods (NRs). Two bands characterize the surface plasmon absorption spectra of AuNRs. The transverse surface plasmon resonance, which is positioned at approximately 520 nm, is responsible for the shorter wavelength band. The shift is to a shorter wavelength, leading to an increment in the nanorod’s average aspect ratio. However, the maximum absorption band is only evident at extended wavelengths, which corresponds to the redshift of longitudinal surface plasmon resonance with an increase in aspect ratio [21,22,23].

Anthracycline doxorubicin (DOX) is effective in treating a wide variety of cancers, including metastatic breast carcinoma and cancers of the blood or lung as well as ovarian and sarcoma. A variety of cancers have been treated using DOX. Using DNA chain-breaking for replication, DOX stops replication [27,28]. It does this by breaking up the DNA chain and preventing it from being replicated [29,30]. To create nano-transporters or nanocarriers for targeted drug delivery systems (DDS), the use of nanotechnology is essential. As a result of their tiny size, these nanocarriers have favorable physiochemical and biological properties [31]. Accordingly, they are very powerful carriers of targeted DDS that successfully transport drugs towards a specific biological location. These nanocarriers are widely used in DDS because of their increased bioavailability [32], active surface area [33], solubility in biofluids [34], rapid anti-cancer therapeutic efficacy [35], and decrement in the dose [36]. Dendrimers, metallic nanoparticles (such as gold nanoparticles), quantum dots, polymeric nanomaterials, and liposomes have all been used previously as nano-transporters in targeted DDS [37].

Jana et al., [26] devised a multistep technique for growing NRs from seeds. The yield of NRs produced by this technique was so low that centrifugation was required to collect them. By adding different amounts of silver ions, they altered the nature of growth solutions. When different amounts of seed solution are added, NRs with different aspect ratios are created. Up to 40–50 percent of the gold nanoparticles generated by this technique are spherical. The formation of particles of φ-shape in this process alters the nanorods’ plasmon absorption bands. In this study, the numerical analysis of electric field enhancement and optical properties of gold nanorods was conducted for biological applications. For experimental study, nanorods were produced through the seed-mediated growth method [38,39]. Later, doxorubicin was used for targeted drug delivery via AuNRs.

## 2. Numerical Analysis

The interaction of gold nanorods with light was studied using the COMSOL Multiphysics tool. The geometry of the problem was discretized using an extremely fine mesh as part of the numerical modelling method. The governing electromagnetic equations for mesh elements were finalized and the elements were then assembled to solve the problem. The finite element method was adopted to solve the system of equations. The numerical simulations were performed through the RF module in COMSOL Multiphysics. A perfect matching layer 10 times larger than the nanorod length was selected to contain the simulation volume and eliminate unwanted reflections from the surroundings. The well-defined boundary conditions were imposed to study the optical behavior of the gold nanostructures. For gold nanorods, the aspect ratio varied from 2.5–4.5, as reported in the published literature [38,39]. In the sensing performance of gold nanorods, the aspect ratio is crucial. The dielectric characteristics of gold nanorods were modelled using data from the study by Johnson and Christy [40].

**Figure 1:**
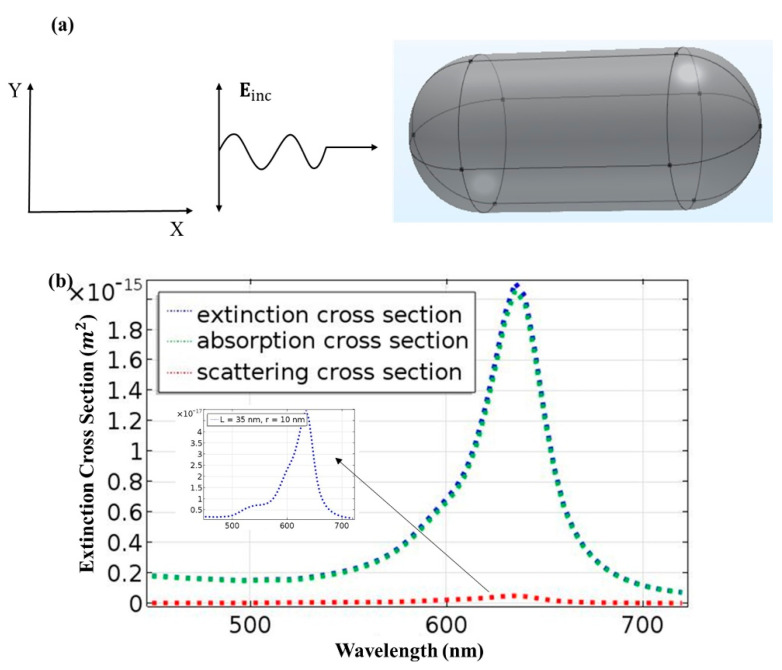
(**a**) Schematic of interaction of an electromagnetic wave with a gold nanorod and (**b**) extinction cross-section of AuNR at λ = 635 nm, length = 35 nm and r = 10 nm.

A gold nanorod with a length of 35 nm and a radius of 10 nm was assumed in the first step. The surrounding medium had a refractive index of 1 and was free space. Figure 1a shows the interaction of light with a gold nanorod (AuNR), while Figure 1b shows a resonance peak at 635 nm for AuNR. The nanorods revealed high functionalization and surface plasmon resonance in the infrared region of the light spectrum.

**Figure 2:**
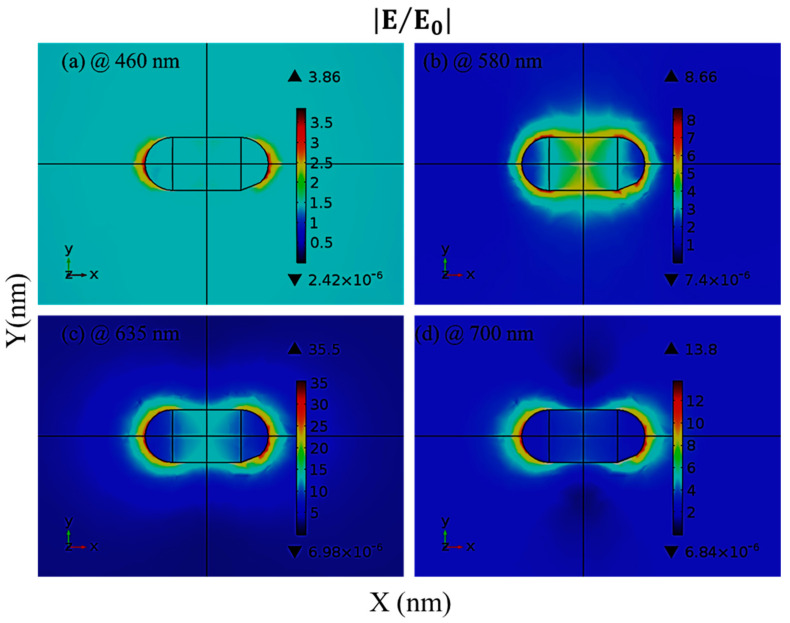
(**a**) Electric field enhancement (V/m) (**a**) at λ = 460 nm, (**b**) at λ = 580 nm, (**c**) at plasmonic resonance (λ = 635 nm) and (**d**) at λ = 700 nm for the gold nanorod of 35 nm length under p-wave illumination.

Figure 2 shows the electric field enhancement $\left|E/E_{0}\right|$ of AuNRs at different resonance wavelengths in the visible part of the light spectrum, which also includes the resonance wavelength of λ = 635 nm. In all cases, it is observed that the highest electric field enhancement lies on the interface between the AuNR and the surrounding medium. As the distance from the interface increases, the electric field decays exponentially. At a wavelength of 460 nm, the electric field enhancement of AuNRs with a length of 35 nm and a radius of 10 nm was determined to be 3.86 at the interface, as illustrated in Figure 2a. The electric field enhancement was 8.66 when the operating wavelength was 580 nm, as shown in Figure 2b. Similarly, Figure 2c is based on Figure 1b, where the operating wavelength was chosen as 635 nm (resonance wavelength) and a maximum electric field enhancement of 35.5 was observed. In the last case in Figure 2d, the wavelength of the incident wave was chosen as 700 nm and 13.8 times higher electric field enhancement was calculated for this wavelength. These findings show that the electric field reaches its highest magnitude at a resonance wavelength of 635 nm. Due to the electric dipole orientation along the X-axis of varied amplitudes, depending on the kind of material and wavelength, the field map revealed a typical two-lobe distribution for all wavelengths.

Gold nanorods are good candidates for electric field enhancement. The use of this feature is important in surface-enhanced molecular sensing and Raman spectroscopy. It is worth noting that the nanorods’ electric field is proportional to their heat-generating capacity. The strength of the electric field within the nanorods is thus directly related to the Joule effect. With a shift in the observation point away from the interface, the augmented electric field decays rapidly. These results imply that the temperature of the nanorod may be controlled by changing the wavelength of incident light. Conclusively, AuNRs are good absorbers of light. This property promotes them as a good candidate for treating tumorous cells.

**Figure 3:**
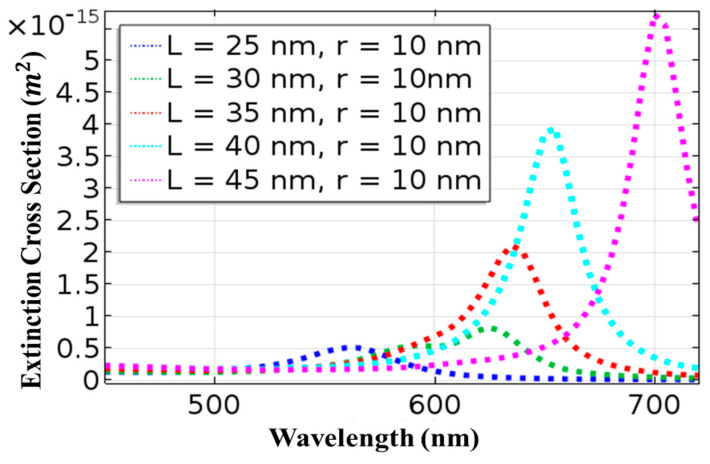
Extinction cross-section of gold nanorods under p-wave illumination.

To explore the optical properties of AuNRs, the extinction cross section, which is the sum of the scattering and absorption cross sections, can be used. Figure 3 shows the resonance peak of an AuNR with a length of 25 nm and a radius of 10 nm at a wavelength of 560 nm. With an increment in nanorod size, the resonance peak exhibits a shift towards a longer wavelength. With an increase in the length of AuNR from 25 to 45 nm while maintaining the radius constant, the resonance peak is shifted from 585 to 700 nm. A difference of 140 nm is reported in Figure 3. It shows that a shift in nanorod size influences both absorption and scattering cross-sections. For example, when the diameter of nanorod is below 70 nm, ∆abs dominates ∆sca, but when diameter is greater than 70 nm, ∆sca dominates ∆abs [41].

**Figure 4:**
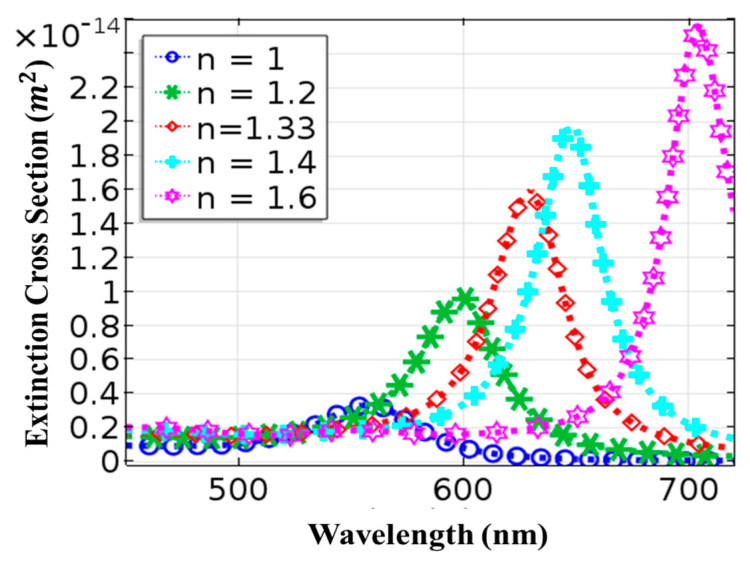
Change in extinction cross-section of the gold nanorods with incident wavelength for different refractive index values.

Another key parameter that affects the value of resonance wavelength is the surrounding medium. For identically sized gold nanorods, the resonance peak amplitude redshifts as the refractive index of the surrounding medium rises. As a result, we fixed the nanorod’s parameters (length: 25 nm, radius: 10 nm) before varying the refractive index of the surrounding medium. The results are reported in Figure 4. With an increase in the refractive index of the surrounding medium, a redshift in the resonance peak was observed. Theoretical results discussed in Figure 1, Figure 2, Figure 3 and Figure 4 show that AuNRs can be considered as sensors with excellent control by optimizing the input parameters. Based on these findings, we prepared the gold nanorods and applied them for biomedical applications in the following sections. When applications of gold nanorods involve heating, a uniform field distribution is required for uniform heating. One such example is the heat treatment of cancer with nanoparticles [42].

## 3. Experimental Analysis

### 3.1. Materials

L-ascorbic acid and Sodium borohydride (99%) were bought from Sigma-Aldrich. Benzyldimethylammonium chloride hydrate (98%) and hexadecyltrimethylammonium bromide (98%) and acquired from Fluka. Deionized water was used as a solvent in preparing the solutions. The steps involved in the preparation of seed, growth solution and nanorods are explained below.

### 3.2. Preparation of Gold Nanorods

The method of preparation of nanorods involved seed-mediated growth, as explained by Nikoobakht and El-Sayed [2]. They capped seed with hexadecyltrimethylammonium bromide (CTAB). A similar seed solution was prepared for production of AuNRs in this study.

#### 3.2.1. Seed Solution

The solution of 0.2 M CTAB (100 mL) was prepared in distilled water by stirring at room temperature. This solution was mixed with the same amount of 0.0005 M HAuCl_4_. By stirring the solution, 12 mL of ice-cold 0.01 M NaBH_4_ was added to the solution. The resulting brownish, yellow-colored solution was stirred for 15 min by maintaining temperature at 25 °C.

#### 3.2.2. Growth Solution

Firstly, 100 mL of CTAB solution was added separately to 3, 4 and 5 mL solutions of 0.0040 M AgNO_3_. The length of the nanorods changes with a change in the amount of AgNO_3_. Then, 100 mL solution of 0.0010 M HAuCl_4_ was prepared and mixed with 1.4 mL of 0.0788 M ascorbic acid. Dark yellow growth solution turned to colorless when ascorbic acid was added as a reducing agent.

#### 3.2.3. Growth of Nanorods

In the last step of the growth process, the growth solution was mixed with 0.34 mL of seed solution. The solution changes its color withing 10–20 min for shorter nanorods. This shift in color was relatively slower for longer nanorods. A constant temperature of 30 °C was maintained during the formation of growth in all experiments. The nanorods of varying aspect ratio can be produced by changing the concentration of salts. The amount of salt solutions, used in this study, is reported in Table 1.

### 3.3. Characterization of Nanorods

The nanorods were washed and dried for further characterization. Scanning electron microscopy, X-ray diffraction spectroscopy and UV-visible spectroscopy tools were considered to elaborate the surface morphology, structural formation and optical properties of the product. The UV vis spectra were generated using a photo spectrophotometer. The baseline setting was done by using ultrapure water. The other measurements were the maximum wavelength and plasmonic resonance band. The structural formation and planes of the produced nanorods were studied using XRD spectra. The length, diameter and surface morphology were studied by producing SEM micrographs of the nanorods. These parameters were compared with the theoretical findings reported in earlier sections.

### 3.4. Discussion of Experimental Results

**Figure 5:**
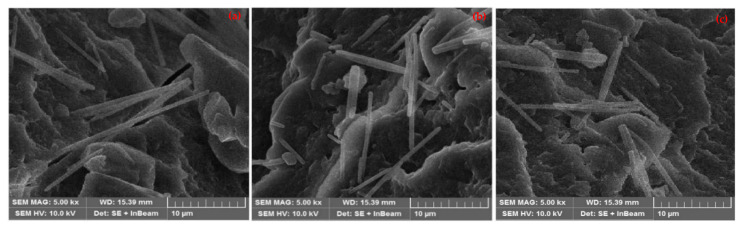
SEM images of AuNRs produced with (**a**) 3 mL of AgNO_3_, (**b**) 4 mL of AgNO_3_, and (**c**) 5 mL of AgNO_3_.

Three samples of AuNRs were prepared by changing the amount of AgNO_3_ solution in the seed-mediated technique. The length of AuNRs changes with a change in the amount of AgNO_3_ solution. Figure 5 shows SEM images of AuNRs produced with 3 mL, 4 mL and 5 mL of AgNO_3_. The rod-like structures with a circular cross-section were seen in all SEM images. However, the uniformity in cross-section and length does not remain the same in all samples. The length of nanorods exhibited an inverse relationship with the concentration of AgNO_3_. The diameters of nanorods were slightly larger and varying in the case of larger concentrations. The smaller concentrations revealed more uniform diameters and lengths of the nanorods. The average length of AuNRs was measured at about 22 nm, 35 nm and 47 nm for 3 mL, 4 mL and 5 mL of AgNO_3_, respectively. The diameter and length were more uniform when the concentration of AgNO_3_ was low. The diameter distribution was wider when AgNO_3_ concentration was high [43]. The length of nanorods decreases as the concentration of silver ions increases. This drop could be owing to silver ions’ high ionic strength. A decrease in the length of nanorods may also be caused by an increase in gold ion concentration. The gold ions and surfactant combine to produce a dark yellow complex, which precipitates as the gold ion concentration rises. The charge density, rod size, and reactivity of the CTAB head group with the gold surface may all alter as a result of this interaction. The rod length and diameter were measured using the built-in tool in SEM imaging software. The aspect ratio was obtained by dividing length by diameter of the nanorods. The average aspect ratio remained between 4.2 and 9.7, depending on the amount of AgNO_3_. The elements in SEM images other than nanorods are referred to as base material. Since the nanorod samples were collected on glass slides and dried for characterization, the solution also dries on the slide along with nanorods. This solution formed the background of the nanorods.

**Figure 6:**
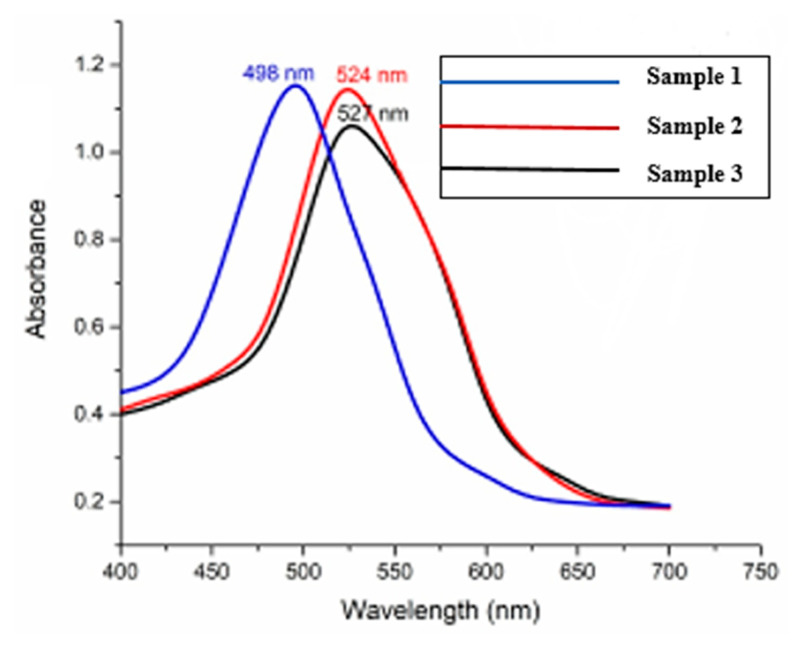
UV-vis spectra of AuNRs produced with 3 mL, 4 mL and 5 mL of AgNO_3_ solution.

Figure 6 reports resonance peaks in UV vis spectra of the nanorods, which appeared due to the extinction cross-section of AuNRs. The UV absorbance increases with the concentration of AgNO_3_ from 3 mL to 5 mL. As the rod diameter increases, the bandgap of the nanorods decreases. These findings suggest a redshift in the transverse plasmon band, which indicates an increase in the aspect ratio of AuNRs. The transverse plasmon band is recorded at 498 nm, 524 nm, and 527 nm for 3 mL, 4 mL and 5 mL of AgNO_3_, respectively. The FWHM of AuNRs increases with a change in the amount of silver nitrate. The UV results show that as we increase the amount of AgNO_3_, there is a redshift in the wavelength. It confirms better optical properties of AuNRs in the visible part of the light spectrum [43,44,45].

**Figure 7:**
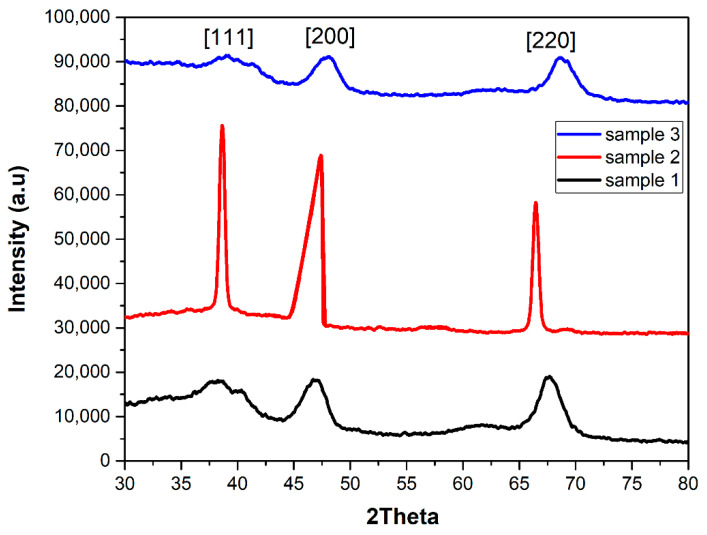
XRD spectra of AuNRs produced with 3 mL, 4 mL and 5 mL of AgNO_3_ solution.

The chemical composition and crystal phases of the samples were investigated further using XRD analysis. Representative XRD patterns of the nanorods, produced with different concentrations of AgNO_3_, are shown in Figure 7. The patterns revealed the formation of the face-centered cubic structure of gold, as confirmed by the PDF 04–0784 ICDD card. Three diffraction peaks were observed in each XRD pattern at 2θ of 38.24°, 46.52°, and 67.66°. The XRD peak intensity changes with seed-mediated growth, as explained by Nikoobakht and El-Sayed [2]. The intensity of the XRD peak corresponding to the (2 0 0) plane was slightly higher than that of the other XRD peaks. These results agree well with the findings of Teo et al. [46]. The prepared AuNRs with absorption maxima of 700 nm by using the seed-mediated growth method. The relative intensity ratio of (2 0 0) and (1 1 1) planes was much higher than the standard value of 0.52. It suggests that the (2 0 0) plane is the major orientation in gold nanostructures. This orientation results in the formation of rod-like gold crystals along the (1 0 0) plane.

### 3.5. Drug Loading and Releasing

**Figure 8:**
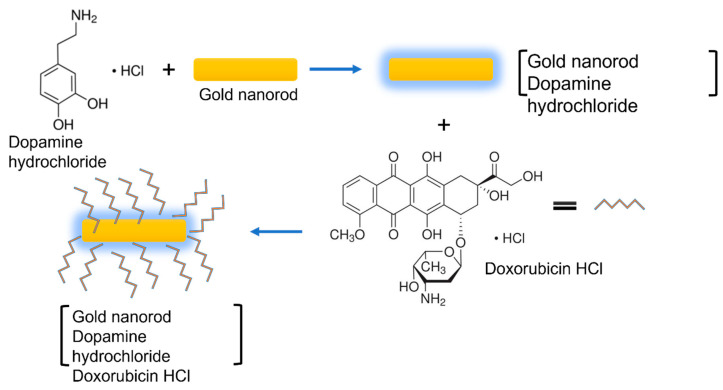
Illustration of the drug loading mechanism.

Doxorubicin (DOX) destroys cancer-infected tissues and cells at all stages of their lifespan. DOX stops the cellular duplication cycle of tumor cells by breaking the DNA sequence that is used for duplication. Irrespective of its widespread utilization, DOX may have adverse effects on the patient’s health due to the release of DOX in the wrong areas rather than on specific diseased tissues. The presented targeted drug delivery system involves two major processes: the loading of the drug on the prepared gold nanocarriers and the release of the loaded drug from the nanocarriers. The drug loading mechanism is illustrated in Figure 8. 

A beaker was filled with 100 mL of distilled water and placed on a magnetic stirrer. A phosphate buffer saline (PBS) tablet of pH 7.4 was added to the solution and stirred for 15 min. The buffer was added to change the pH of the solution and imitate the mechanism of the human body. Then, 0.2 g of AuNRs was added to the solution under constant stirring. These nanorods were coated with a biocompatible layer of a neurotransmitter (Dopamine hydrochloride). About 5 mL of Dopamine HCl was added to the solution and stirred vigorously. Thereafter, 2.5 mL of DOX was added to the solution. Then, 2 mL of solution was collected for UV-Vis analysis after regular intervals of time. The same amount of fresh PBS was added to the solution each time.

**Figure 9:**
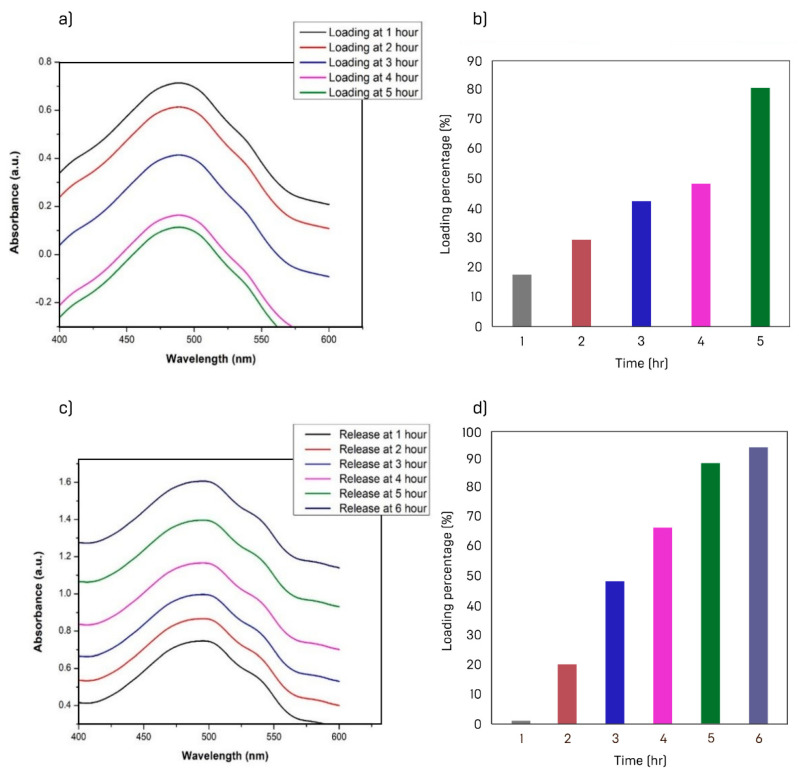
(**a**) Change in UV absorbance with loading time; (**b**) bar graph shows the decrease in loading capacity with time; (**c**) change in UV absorbance with time during desorption; (**d**) bar graph of desorption with time.

After drug loading, the release was carried out through temperature stimuli or a heat triggered drug release system. The nanoparticles, loaded with drug, were stirred continuously for one hour by maintaining the temperature at 35 °C. Then, 2 mL of the solution was collected for UV-Vis analysis. The temperature was then raised by 5 °C under continuous stirring for 1 h in the dark environment. The sample was collected for UV-Vis spectroscopy before raising the temperature further by 5 °C. The maximum absorbance of AuNRs solution is seen after 1 h of incubation time, which started to decrease with the further increase in incubation time. The minimum absorbance is observed after 5 h of incubation. The drug loading capacity increased with the passage of time. Roughly, an 80% increment in load capacity was observed in Figure 9b. For desorption of the drug, a pH 3.7 buffer solution of phosphate was used. The absorbance of the solution increased with time from the occurrence of the desorption of doxorubicin. This indicates that the desorption process led to an increase in the drug’s concentration, as portrayed in Figure 9c. Desorption of the solution showed an increasing trend after the first hour of incubation and up to 6 h. About 93% of drugs were released after 6 h of desorption, which showed the excellent ability of AuNRs for time-dependent drug delivery.

**Figure 10:**
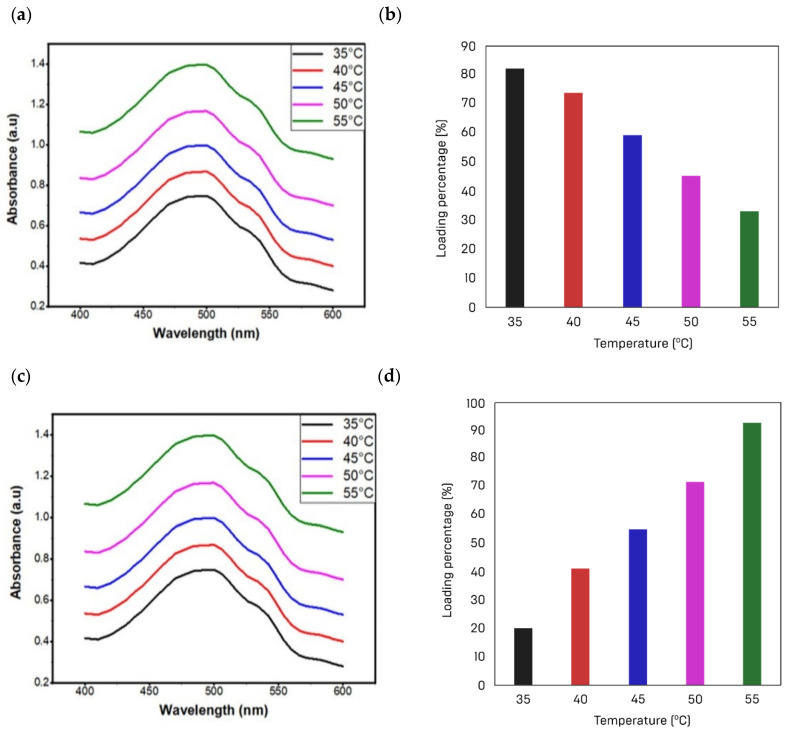
(**a**) Variation in UV absorbance as a function of loading temperature; (**b**) a bar graph illustrates the reduction in loading capacity over temperature; (**c**) during desorption, the UV absorbance changes with temperature; (**d**) pattern of desorption over temperature is illustrated as a bar graph.

Five solutions of doxorubicin medicine of the same concentration were mixed with 0.5% of AuNRs@DA at different temperatures. The objective was to find out the appropriate drug loading temperature. The drug loading capacity started to decline with a rise in temperature. A temperature of 25 °C was found optimum for drug loading capacity under the investigated conditions. The drug loading efficiency decreases as the temperature rises, as seen in Figure 10. This trend reveals that higher temperatures are not suitable for better drug loading with AuNRs@DA. These results agree well with the results reported in the previous studies, as shown in Table 2.

**Figure 11:**
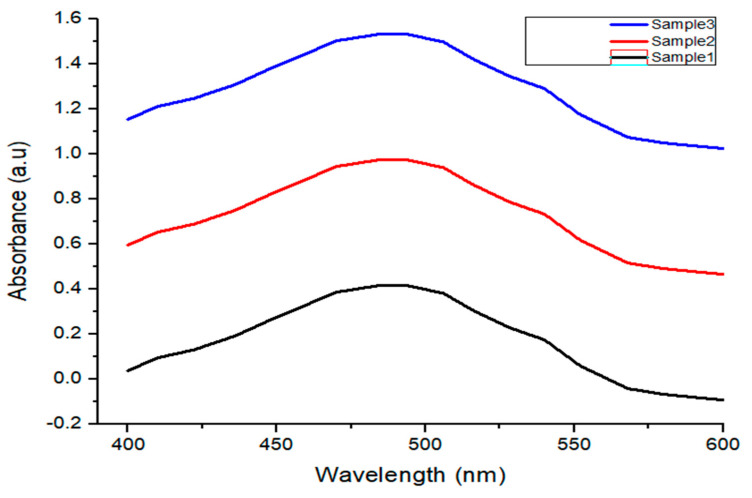
UV–Vis spectra of solutions prepared with different amounts of AuNPs (0.3, 0.4, 0.5 g).

Contrary to the loading capacity, the release of the drug increased proportionally with a rise in temperature. The release of drug is also assessed in PBS at several temperatures (45, 40, 30, 20, and 25 °C) by fixing the solution pH at 7.4. The drug desorption was measured to be about 93% at a temperature of 45 °C. As the temperature increases, the drug easily desorbed off the nanorods, as shown in Figure 10c,d. This property made it suitable material in combating cancer cells. Another important component of a targeted drug-delivery system is the number of drug carriers. Different quantities of nanorods (0.3, 0.4, 0.5 g) were mixed with the drug solution to test the effect of the number of nanorods on drug incubation. A UV-Vis spectrophotometer was used for measuring the light absorbance by the solution. The absorbance data show that the number of active sites increases with the number of nanorods. The drug loading capacity increases with a rise in active sites, as shown in Figure 11. In this study, 0.4 g of nanorods was suggested for drug delivery applications.

## 4. Conclusions

In this study, analytical and experimental methods were used to study the potential application of gold nanorods in drug delivery for cancer treatment. COMSOL Multiphysics was used to investigate the electric field enhancement and optical properties of AuNRs for different incident wavelengths. The theoretical results suggest the good suitability of the produced gold nanorods for anticancer applications. Morphological and optical characteristics of AuNRs were evaluated through XRD, SEM and UV–visible analysis. The relative intensity ratio of the planes (2 0 0) and (1 1 1) was significantly higher than the usual value of 0.52. It suggests that, in gold nanostructures, the (2 0 0) plane is the dominant orientation. The production of rod-like gold crystals along the (1 0 0) plane occurs as a result of this orientation. Later, gold nanorods were applied for the targeted delivery of doxorubicin medicine. The maximum absorbance of AuNRs solution was observed after 1 h of incubation time, which started to decrease with a further increase in incubation time. The minimum absorbance was observed after 5 h of incubation. Desorption of the solution showed an increasing trend after the first hour of incubation and up to 6 h. The release of the drug increased proportionally with a rise in temperature. The drug desorption was measured to be about 93% at a temperature of 45 °C. Finally, 0.4 g of nanorods was suggested for drug delivery applications.

## Figures and Tables

**Table 1:** Chemicals and their amount used for preparation of AuNRs.

Chemicals	Sample 1	Sample 2	Sample 3
CTAB	100 mL	100 mL	100 mL
AgNO_3_	3 mL	4 mL	5 mL
HAuCl_4_	100 mL	100 mL	100 mL
Ascorbic Acid	1.4 mL	1.4 mL	1.4 mL
Seed Solution	0.34 mL	0.34 mL	0.34 mL

**Table 2:** Comparison of drug loading and releasing for different compositions of AuNRs.

Composition	Drug Loading Percentage	Drug Releasing Percentage	Reference
Au/SiO_2_/HAP	98.89 ± 0.6%	>95%	[47]
DOX-PSS-GNRs	76%	99%	[48]
GNR-PDA	52.6%	88%	[49]
GNRs@DA	80%	93%	Present Study

## Data Availability

The data is available from the corresponding author on reasonable request.
